# Case Report: Cardiotoxicity of capecitabine may manifest as STEMI with significant left ventricular cardiac dysfunction and recurrent supraventricular and ventricular arrhythmias: a proposal for optimal diagnosis and treatment

**DOI:** 10.3389/fonc.2025.1576415

**Published:** 2025-06-06

**Authors:** Sebastian Szmit, Małgorzata Wojciechowska, Barbara Sobera, Krystian Szczypiorski, Izabela Poprawa, Dagmara Gralak-Łachowska, Maciej Zarębiński

**Affiliations:** ^1^ Department of Cardio-Oncology, Centre of Postgraduate Medical Education, Warsaw, Poland; ^2^ Department of Cancer Diagnostics and Cardio-Oncology, Maria Sklodowska-Curie National Research Institute of Oncology, Warsaw, Poland; ^3^ Chair and Department of Experimental and Clinical Physiology, Laboratory of Centre for Preclinical Research, Medical University of Warsaw, Warsaw, Poland; ^4^ Department of Invasive Cardiology, Independent Public Specialist Western Hospital John Paul II, Lazarski University, Grodzisk Mazowiecki, Poland

**Keywords:** colorectal cancer, capecitabine, myocardial infarction, atrial fibrillation, cardiac arrest

## Abstract

Colorectal cancer is one of the most prevalent cancers globally, representing approximately 10% of all cancer cases. Due to its prevalence, an important issue is the cardiotoxicity of chemotherapy used in the course of the disease. In this article, we present the case of a patient with sigmoid cancer T3N1M0 and rectal cancer T2N0M0 who started postoperative chemotherapy according to the XELOX (CAPOX) regimen (oxaliplatin with capecitabine). A few days later, he experienced chemotherapy-related myocardial injury, which presented clinically as an ST-segment elevation myocardial infarction and was further complicated by atrial fibrillation, a severe ventricular arrhythmia, and cardiac arrest. Urgent angiography excluded significant changes in the coronary vessels, but a marked reduction in left ventricular systolic function was observed in echocardiography. Takotsubo syndrome and myocarditis were included in the differential diagnosis. Finally, a transient coronary artery spasm was deemed the most probable cause, as temporary ST-segment elevation episodes were noted on the ECG in the initial days of hospitalization. Cardiological treatment resulted in significant improvement of the clinical condition, including improvement of left ventricular systolic function and cessation of arrhythmias. Immediately after leaving the hospital, the patient received LifeVest for the prevention of sudden death and then was qualified for implantable cardioverter–defibrillator (ICD) implantation. The importance of the above adjuvant chemotherapy for the prognosis has been confirmed by the fact that after 1 year of observation, the patient experienced a cancer relapse with metastases to the lungs and peritoneum. *Conclusions*: This case highlights that severe cardiovascular toxicity from cancer treatment remains a significant issue, critically affecting patient prognosis. Identifying predictors of such complications is essential to enable early prevention. An alternative approach may involve the development of novel anticancer treatments with reduced cardiotoxicity.

## 1 Introduction

Fluoropyrimidines, both intravenously administered 5-fluorouracil (5-FU) and orally administered capecitabine, are characterized by cardiovascular toxicity (1, 2). The most common clinical manifestation is chest pain resulting from a vasospasm reaction. This complication leads to myocardial ischemia and can be associated with serious consequences such as ventricular arrhythmias and sudden cardiac arrest. It is possible, however, that the vasoconstrictor effect of the drug is superimposed on other mechanisms of cardiovascular toxicity.

The 2022 European Society of Cardiology (ESC) guidelines on cardio-oncology include a dedicated chapter addressing complications related to fluoropyrimidines (3). Fluoropyrimidine-induced cardiotoxicity reflects the full spectrum of cancer therapy-related cardiovascular toxicity (CTR-CVT) (4), ranging from classic manifestations, such as angina pectoris and acute coronary syndrome, to less common presentations including heart failure, arterial hypertension, Takotsubo syndrome, myocarditis, various arrhythmias, and even ischemic stroke (5). Cardiac ischemia may affect up to 10% of patients receiving fluoropyrimidine-based chemotherapy, and the most classic pathomechanism is considered to be acute vascular spasm and endothelial damage (6, 7). Naturally, in every case of chest pain with troponin release and ECG changes, before ultimately diagnosing chemotherapy-induced coronary artery spasm, it is essential to exclude type 1 myocardial infarction, Takotsubo syndrome, and myocarditis, bearing in mind that all three of these conditions could be entirely unrelated to cancer treatment (e.g., Takotsubo syndrome may be associated with stress related to the illness itself). However, even a small degree of vasospasm reaction becomes clinically significant in patients with concomitant atherosclerosis (2). Patients with coexisting heart disease have a 5.5 times greater chance of cardiotoxicity (8–10), but severe cardiac injury may occur even in young individuals without evidence of atherosclerotic plaques. In such cases, heart failure is attributed not primarily to myocardial ischemia but rather to direct toxic insult to cardiomyocytes (11). Among those without heart disease, hypercholesterolemia, arterial hypertension, and current smoking remain significant risk factors for cardiotoxicity of fluoropyrimidines.

We present a patient with severe complications during chemotherapy with capecitabine used as an adjuvant therapy after surgical treatment of colorectal cancer. The report highlights the importance of dynamic differential diagnosis and effective cardiac pharmacotherapy.

## 2 Case presentation

The patient was a 65-year-old white man of Central European (Polish) descent who was overweight (body mass index (BMI) 26.2 kg/m^2^), had no hereditary background of cardiovascular disease, had a history of untreated arterial hypertension, and declared to have never smoked. He underwent a sigmoid and upper rectal resection on June 27, 2023. Postoperative histopathological evaluation revealed sigmoid G1 adenocarcinoma with features of periintestinal tissue invasion pT3, without vascular and perineural invasion, in two of 18 assessed lymph nodes, and the presence of neoplastic disease was recognized as pN1b. The second diagnosis was rectal adenocarcinoma pT2 N0. Based on imaging tests of the chest, abdomen, and pelvis, no distant metastases of cancer were found—M0. The patient was qualified for adjuvant chemotherapy according to XELOX (CAPOX) regimen for 6 months: oxaliplatin 130 mg/m^2^ at 2-hour intravenous infusion on day 1 and then capecitabine (Xeloda) with everyday dose of 1,000 mg/m^2^, which means a patient daily dose of 4,000 mg for 14 days (twice daily 4 tablets of 500 mg) and then a 7-day break. On September 18, chemotherapy was started.

**Figure 1:**
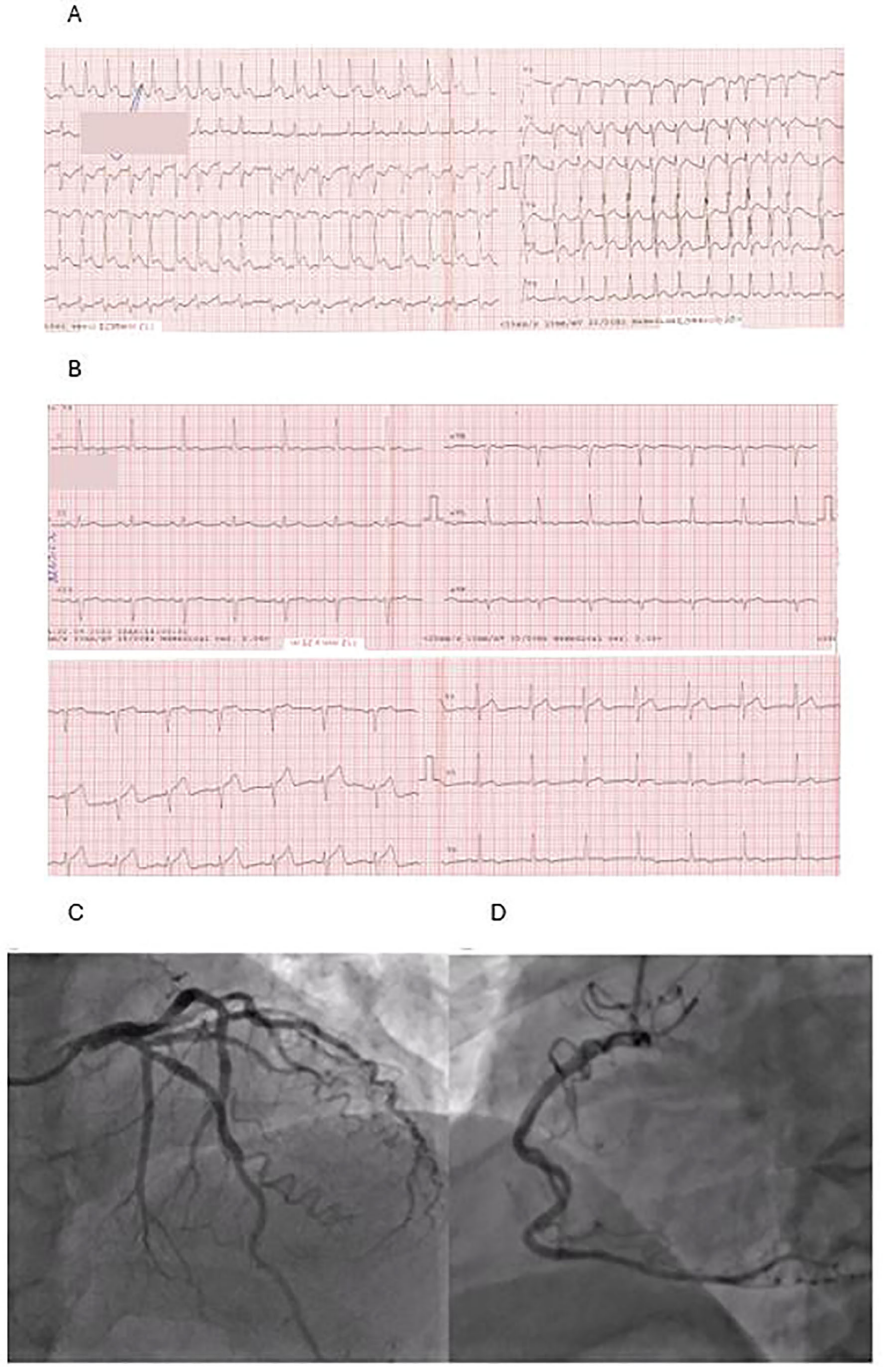
**(A)** ECG taken during the first contact with medical staff: atrial fibrillation, ST-segment elevation in leads: I, aVL, V5-V6. **(B)** ECG made on admission: normal sinus rhythm and normalization of ST segment. **(C, D)** Coronarography: left coronary artery **(C)** and right coronary artery **(D)**—coronary arteries without atherosclerotic changes.

Symptoms of severe chest pain occurred 4 days after initiating chemotherapy. The chest pain lasted 2 hours before the patient decided to call for an ambulance. ECG showed the picture of atrial fibrillation and lateral ST-segment elevation myocardial infarction (STEMI) (**Figure 1A**). The patient was saturated with aspirin 300 mg and ticagrelor 180 mg and was given heparin 5.000 IU i.v. during the transport to the hospital, and chest pain subsided.

On admission to the hospital, the patient was in Killip class 1 with blood pressure 180/90 mmHg and a heart rate of 70 beats/min. Repeated ECG revealed sinus rhythm and normalization of the ST segment (**Figure 1B**). Urgent coronarography showed no significant coronary artery disease (**Figures 1C, D**). The echocardiogram demonstrated left ventricular systolic dysfunction with apical akinesis and left ventricular ejection fraction (LVEF) of 35%, with no other relevant findings. The results of the laboratory tests made on admission are shown in **Table 1**.

**Table 1:** Patient’s test results at admission (only Troponin Ths has a maximum value recorded during hospitalization) and the drugs prescribed at discharge from the hospital.

Parameter [units] (reference range)	Result
RBC [10^6^/µl] (3.7–4.8)	5.47
WBC [10^3^/µL] (4.0–10.0)	6.07
Hb [g/dL] (12.2–15.8)	13.5
MCV [fL] (80-96)	78.4
PLT [10^3^/µL] (130–400)	187
Troponin Ths [ng/mL] (0–0.014)	0.090
Creatinine [mg/dL] (0.70–1.2)	0.92
Sodium [mmol/L] (132–146)	144
Potassium [mmol/L] (3.7–5.4)	3.6
Glucose [mg/dL] (70–99)	153
CRP [mg/L] (0–5.0)	2.0
BNP [pg/mL] (5.0–125)	1232
D-dimer [µg/mL] (0–0.5)	0.71
HbA1c [%] (4.5–5.7)	5.8
LDL (mg/dL)	141
Drug with dosage	Frequency
Amiodarone 200 mg*	QD
Amlodipine 10 mg	QD
Apixabanum 5 mg	BID
Atorvastatin 40 mg	QD
Bisoprolol 2.5 mg	QD
Empagliflozin 10 mg	QD
Eplerenone 25 mg	QD
PPI 40 mg	QD
Ramiprilum 10 mg	QD
Torasemide 5 mg	QD

BID, bis in die (twice a day); BNP, B-type natriuretic peptide; CRP, C-reactive protein; Hb, hemoglobin; HbA1c, hemoglobin A1c; LDL, low-density lipoprotein; MCV, mean corpuscular volume; PLT, platelets; PPI, proton pump inhibitor; QD, quaque die (once a day); RBC, red blood cells; Troponin Ths, troponin T high sensitivity; WBC, white blood cells.

* Amiodarone therapy was recommended for 3 months.

Low-molecular-weight heparin in a therapeutic dose and typical treatment for heart failure with reduced ejection fraction (HFrEF), including beta-blockers, angiotensin-converting enzyme (ACEI) inhibitors, eplerenone, and sodium-glucose co-transporter 2 (SGLT2) inhibitors, were initiated. An electrolyte solution with potassium and magnesium was also given to the patient. Due to the accompanying elevated blood pressure, nitroglycerin infusion was introduced as well. However, during the first 2 days, recurrent episodes of atrial fibrillation were observed. Moreover, the patient presented further episodes of chest pain with ST-segment elevation visible on the cardiac monitor. It is noteworthy that in some of these episodes, ST-segment elevation was immediately followed by ventricular tachycardia, which then quickly progressed to ventricular fibrillation. During the first 48 hours of hospitalization, a few defibrillations were necessary. In ECG recordings performed between arrhythmia episodes, the QT interval remained normal.

Myocarditis was suspected once atherosclerotic changes in the coronary arteries had been ruled out; however, inflammatory markers were low, and the patient had no recent history of infection. The clinical presentation was also not suggestive of Takotsubo syndrome. Ultimately, a transient coronary artery spasm was deemed the most probable cause, as temporary ST-segment elevation episodes were observed on the ECG during the initial days of hospitalization.

Based on the literature concerning capecitabine, its cardiotoxic effects were suspected, especially epicardial artery spasm. Thus, calcium channel blockers were administered (12). Additionally, antiarrhythmic therapy was implemented with amiodarone. All of these resulted in the resolution of the recurrent chest pain and arrhythmia. On the third day of hospitalization, the patient did not report any symptoms, was free from ventricular and supraventricular arrhythmias, and had blood pressure that remained within the normal range.

**Figure 2:**
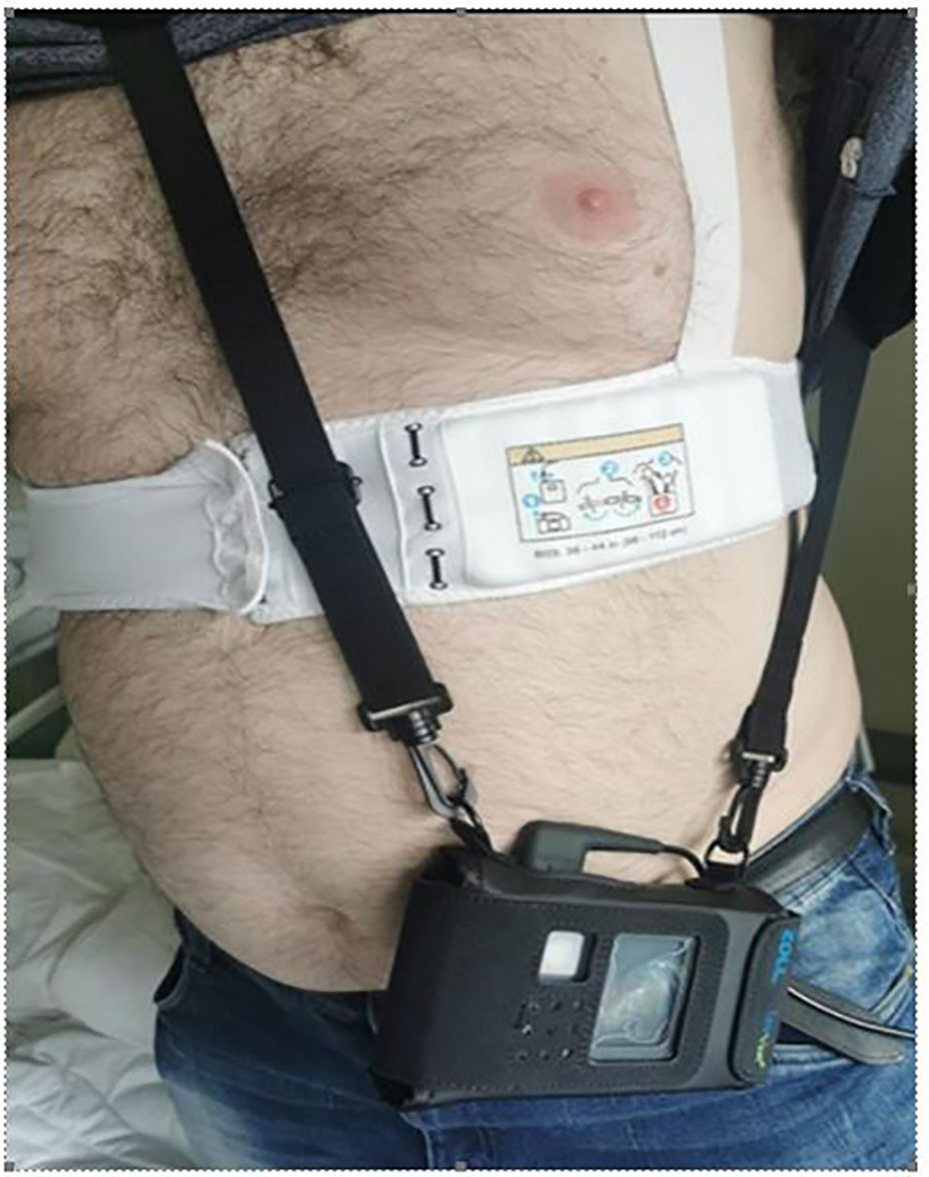
Patient secured with LifeVest.

The patient was discharged after 8 days of hospitalization and secured with LifeVest (**Figure 2**). Control echocardiogram before discharge showed LVEF of 45%. As MI was secondary to vasospasm, considering the recurrent attacks of atrial fibrillation and recent history of colorectal cancer, the patient received non-vitamin K antagonist (VKA) oral anticoagulant but no antiplatelet treatment. The list of medications prescribed for the patient is included in **Table 1**.

During the 3-month follow-up, there were neither further episodes of chest pain nor arrhythmia. After that period, LVEF increased to 50%. However, in accordance with the guidelines and after discussing the problem with the patient, it was decided to implant an implantable cardioverter–defibrillator (ICD) for secondary prevention of cardiac arrest. Due to concerns about severe life-threatening side effects, the adjuvant chemotherapy was not continued.

### 2.1 Patient’s perspective and follow-up

After a severe cardiac episode, the patient continued cardiac therapy and took all the recommended medications. In a follow-up echocardiography examination, EF remained approximately 50%. However, the patient stopped seeing an oncologist for the control of the cancer. After only 1 year, when he started feeling unwell, he decided to report to an oncology center. Computed tomography revealed a cancer recurrence in the form of metastatic lesions in the peritoneum and lungs. The oncology team recommended a chemotherapy regimen excluding fluoropyrimidines, which the patient accepted. He started palliative chemotherapy with irinotecan in September 2024, exactly 1 year after he received adjuvant chemotherapy with capecitabine and oxaliplatin. The first two chemotherapy administrations took place in a hospital setting with constant ECG monitoring. Chemotherapy was continued until April 2025 (7 months). In the last CT scan, the changes in the lungs were stable; however, a suspicious lesion appeared in the liver. During the most recent ICD check-up in March 2025, the device memory showed a few isolated episodes of non-sustained ventricular tachycardia (nsVT) with a maximum of 3 beats.

## 3 Discussion

The patient described above had a diagnosis of stage III colon cancer and, after radical surgical treatment, had indications for adjuvant chemotherapy including fluoropyrimidines with oxaliplatin (13). The CAPOX (XELOX) regimen is a valuable option for this indication, recommended for a period of 3 or 6 months (14). Shortening the duration of adjuvant therapy is aimed at eliminating toxicity, especially neurological toxicity.

Cardiovascular toxicity of the FOLFOX (5-FU and oxaliplatin) or CAPOX regimen is primarily related to the risk of acute coronary syndromes (15). Oxaliplatin may provoke type 1 myocardial infarction (4). However, oxaliplatin alone may also trigger STEMI via coronary artery spasm (9). 5-FU in the FOLFOX regimen and capecitabine in the CAPOX regimen rather induce type 2 myocardial infarction in the course of coronary vasospasm (10, 16, 17). Interestingly, those experiencing coronary vasospasm with 5-FU may likewise be at risk of vasospastic events when treated with capecitabine (10).

Depending on the dose, cardiac side effects related to fluoropyrimidines occur within 24 hours after drug administration (18, 19). The clinical picture of their cardiovascular toxicity may be multifactorial, and in addition to the vasospasm mentioned above, other complications may also occur. These are cardiomyopathy, arrhythmias (atrial fibrillation and ventricular arrhythmias), QT prolongation, arterial hypertension, and thromboembolic events (11, 20). Episodes of cardiogenic shock have also been reported (21), along with documented cases of Takotsubo syndrome and myocarditis (22–24).

**Figure 3:**
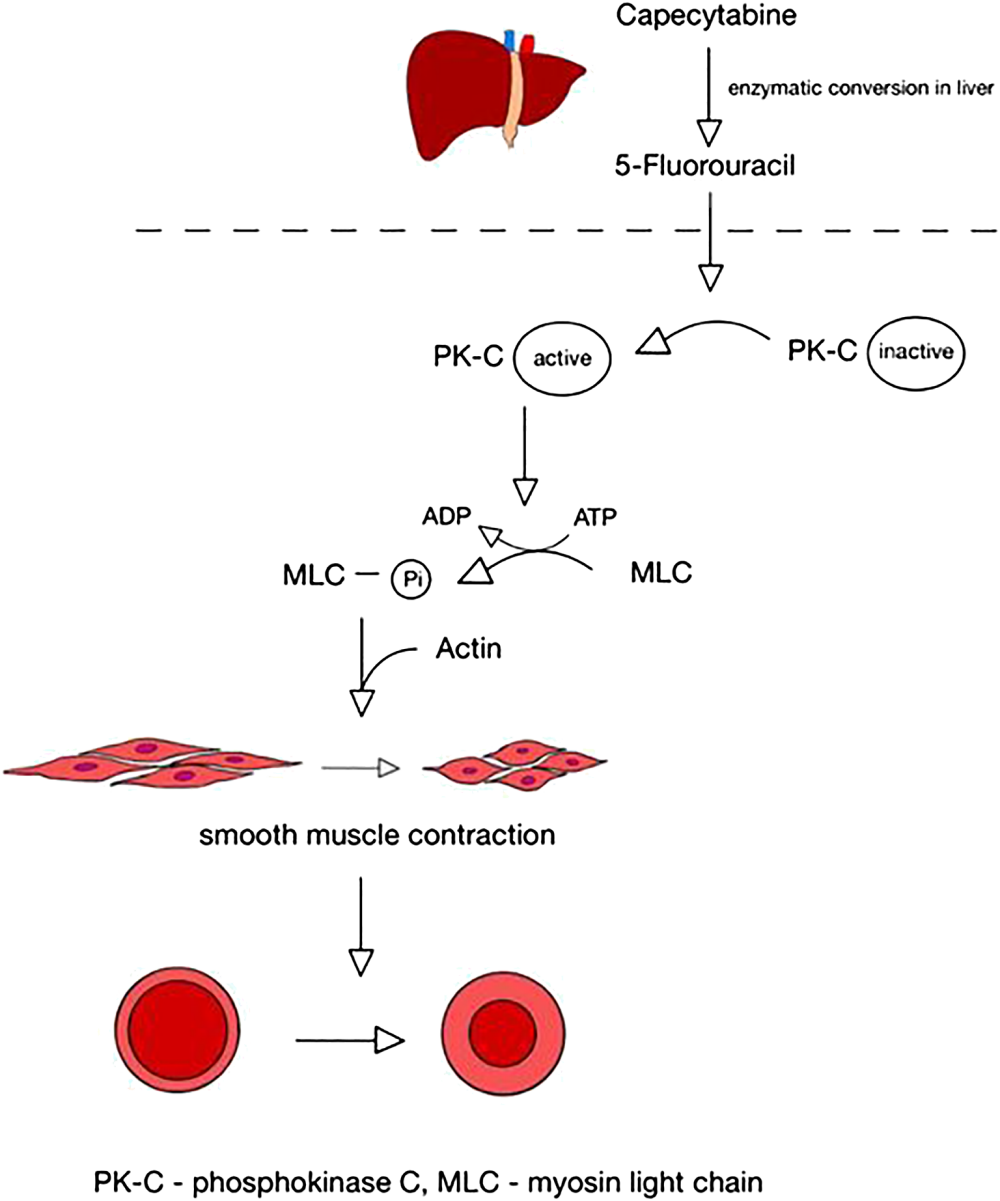
Possible mechanisms of capecitabine-induced vasospasm.

Capecitabine is an orally administered prodrug of 5-FU. The direct mechanism of action of capecitabine leading to vasoconstriction is not well-documented in the literature (2). However, the role of its main metabolite 5-FU in causing vasoconstriction has been proven. The research showed that 5-FU directly activates the protein kinase C (PK-C), which is responsible for the phosphorylation of the light chains of myosin in smooth muscle cells, causing muscle spasm (**Figure 3**). This mechanism of vasoconstrictive potential of 5-FU is shown to be independent of vasoactive membrane receptors and phosphoinositide turnover or cyclooxygenase pathway mediators (25). There are also different indirect pathways through which it may exert vasoconstrictive effects. One of them is endothelial dysfunction that leads to decreased production of nitric oxide (NO) and increased production of vasoconstrictors, mainly endothelin 1 (ET-1) (26). Moreover, the drug has pro-inflammatory effects and activates the sympathetic nervous system. On top of that, electrolyte imbalance is observed in the form of hypomagnesemia or hypokalemia.

Patients with even early-stage colon cancer are at increased risk of myocardial infarction, likely due to a pro-inflammatory state and increased blood clotting tendency (27, 28). Experts agree that significant atherosclerotic changes should be subjected to revascularization before starting chemotherapy (2). The European Society of Cardiology guidelines on cardio-oncology recommend assessment of classic risk factors (arterial hypertension, lipid disorders, and HbA1c) and possible assessment by SCORE/SCORE2-OP (3). However, the level of evidence is based only on the opinion of experts. Screening for coronary artery disease may be considered in high- and very-high-risk patients, but how do we categorize patients like the one presented, when the only known risk factors—arterial hypertension and overweight—do not seem to explain the real risk of such severe complications?

Many earlier studies have shown that a higher risk of cardiovascular events was observed in patients with cardiac comorbidities like co-existing ischemic heart disease or left ventricular dysfunction, as well as in those with smoking history, hyperlipidemia, and renal disease. However, more recently, it has been noticed that patients at higher risk of coronary artery spasm are usually younger and have fewer traditional cardiovascular risk factors than those who do not develop the condition. In one of the studies, higher BMI (probably because of higher total chemotherapy doses)? and the use of beta-blockers (possible aggravation of alpha‐mediated vasoconstriction)? defined a patient at risk of cardiotoxicity related to fluoropyrimidine-based chemotherapy (29). It is increasingly recognized that inherited enzyme polymorphisms and variations in metabolic pathways may influence the metabolism of fluoropyrimidines, contributing to individual susceptibility to their toxicity (2).

### 3.1 Implications of practice

The unique value of our paper is in demonstrating the necessity of an optimal diagnostic approach for each patient with acute coronary syndrome and suspected chemotherapy-induced cardiotoxicity. We emphasize the importance of invasive diagnostics, along with an assessment of the need for possible coronary revascularization. We believe that the decisive factor in the patient’s recovery was the administration of a calcium channel blocker and nitroglycerin (both with vasodilatory properties), amiodarone (for its antiarrhythmic action), and optimal heart failure management. Although the severe left ventricular dysfunction and life-threatening arrhythmias may have resolved spontaneously with the clearance of capecitabine metabolites, the patient’s survival was ultimately attributed to defibrillation and the cardiological pharmacotherapy administered.

Rechallenge to fluoropyrimidines after cardiac side effects is under discussion (1). In this case, continuing fluoropyrimidine-based chemotherapy was not an option, as adjuvant anticancer treatment cannot proceed following such a severe cardiac complication. However, our patient had a high risk of recurrence of cancer. Indeed, the disease progressed after 1 year of observation. The multidisciplinary team taking care of the patient faced a difficult decision to consider chemotherapy with fluoropyrimidines for metastatic cancer.

The patient’s medical history and the rapid recurrence of colon cancer support the decision to implant an ICD as fully justified. The device provides protection in case future cancer treatments trigger another episode of dangerous arrhythmia. As a result, we have enabled the patient to pursue alternative oncological treatment options. The question arises whether anyone worldwide has attempted to reintroduce fluoropyrimidine-based chemotherapy after a certain period. Some case reports have described the use of shortened 5-fluorouracil infusions, often administered as a bolus (18). Other authors have opted for dose reductions combined with prophylactic vasodilator therapy. However, to our knowledge, no one has attempted such treatment in the context of complications as severe as those observed in our patient.

Employing a different treatment strategy represents a potential alternative (29). However, all options of chemotherapy recommended by the European Society for Medical Oncology (ESMO) Clinical Practice Guidelines for localized colon cancer are based on fluoropyrimidines, even for stage II, and the intermediate risk de Gramont scheme with 5-FU is recommended (capecitabine may be an alternative choice) (27). First-line therapy for metastatic colon cancer recommended by the ESMO is also mainly based on fluoropyrimidines: monotherapy plus bevacizumab for frail and elderly patients and doublet or triplet chemotherapy with possible biological targeted agents for others (3). Doublet chemotherapy means FOLFOX (5-FU and oxaliplatin) or FOLFIRI (5-FU and irinotecan). Triplet chemotherapy is the combination of 5-FU, oxaliplatin, and irinotecan (FOLFOXIRI). All other choices are non-standard procedures. This only highlights how challenging it is to discontinue fluoropyrimidines due to their toxicity.

Therefore, the best clinical solution would be to identify factors predicting severe cardiac complications related to fluoropyrimidine. It is unlikely that the classic risk factors for atherosclerosis can reliably identify patients who should not receive this type of chemotherapy. Metabolism of fluoropyrimidines is presumed to be the key element in understanding the observed toxicities. Focusing on the metabolic byproducts may reveal underlying enzymatic impairments, indicating patients at increased risk. Therefore, further studies are needed in this area.

### 3.2 Limitations of the observation

This report is based solely on a single case observation, making it difficult to establish any definitive guidelines. Nevertheless, such a severe complication in clinical oncology warrants discussion. As authors, we believe there is a clear need to systematically register such cases. Current literature shows significant discrepancies, and reported fluoropyrimidine-related complications vary widely in their presentation (18, 20). There are limited data regarding the effectiveness of treatments for the cardiovascular toxicity associated with these agents. Evidence on the use of a defibrillating vest is scarce, and the role of an implantable ICD remains unclear (3, 17). Preventive strategies are virtually non-existent. Therefore, we propose that this case serve as a starting point for deeper analysis and a foundation for future research in this area.

## Data Availability

The original contributions presented in the study are included in the article/supplementary material. Further inquiries can be directed to the corresponding author.
